# Combination of MCL-1 and BCL-2 inhibitors is a promising approach for a host-directed therapy for tuberculosis

**DOI:** 10.1016/j.biopha.2023.115738

**Published:** 2023-10-19

**Authors:** Eusondia Arnett, Susanta Pahari, Chrissy M. Leopold Wager, Elizabeth Hernandez, Jordan R. Bonifacio, Miranda Lumbreras, Charles Renshaw, Maria J. Montoya, Joseph T. Opferman, Larry S. Schlesinger

**Affiliations:** a Host Pathogen Interactions Program, Texas Biomedical Research Institute, San Antonio, TX 78227, USA; b St. Jude Children’s Research Hospital, Memphis, TN 38105, USA

**Keywords:** Tuberculosis, Apoptosis, Macrophage, Granuloma, Host-directed therapy, Drug resistant tuberculosis

## Abstract

Tuberculosis (TB) accounts for 1.6 million deaths annually and over 25% of deaths due to antimicrobial resistance. *Mycobacterium tuberculosis* (*M.tb*) drives MCL-1 expression (family member of anti-apoptotic BCL-2 proteins) to limit apoptosis and grow intracellularly in human macrophages. The feasibility of re-purposing specific MCL-1 and BCL-2 inhibitors to limit *M.tb* growth, using inhibitors that are in clinical trials and FDA-approved for cancer treatment has not be tested previously. We show that specifically inhibiting MCL-1 and BCL-2 induces apoptosis of *M.tb-*infected macrophages, and markedly reduces *M.tb* growth in human and murine macrophages, and in a pre-clinical model of human granulomas. MCL-1 and BCL-2 inhibitors limit growth of drug resistant and susceptible *M.tb* in macrophages and act in additive fashion with the antibiotics isoniazid and rifampicin. This exciting work uncovers targeting the intrinsic apoptosis pathway as a promising approach for TB host-directed therapy. Since safety and activity studies are underway in cancer clinics for MCL-1 and BCL-2 inhibitors, we expect that re-purposing them for TB treatment should translate more readily and rapidly to the clinic. Thus, the work supports further development of this host-directed therapy approach to augment current TB treatment.

## 1 Introduction

Tuberculosis (TB) is the number one infectious disease killer in human history, accounting for over 1 billion deaths [1,2]. TB is currently the second leading cause of death from a single infectious agent, second only to COVID-19 in the past two years. Efforts to control TB were stalled, and even reversed, due to the COVID-19 pandemic, with the TB incidence rate increasing by 3.6% between 2020 and 2021, reversing the average 2% decline over the past 20 years. The number of TB deaths also increased from 2019 to 2021, representing the first increase in fatality rate for TB since 2005. Without treatment, the death rate due to TB is 50%. However, TB treatment has an 85% cure rate, and requires a cocktail of rifampicin (RIF), isoniazid (INH), ethambutol, and pyrazinamide for 4–6 months, although there are efforts to shorten this to 1–6 months. Unfortunately, although cases of RIF resistant and multidrug-resistant (MDR; defined as RIF and INH R) TB increased in 2021, the number of people receiving treatment actually decreased. Treatment for drug resistant TB is even more challenging, more costly and associated with more side-effects although new regimens have reduced treatment from 20 + months to 6 months and improved success rates to 60% [1]. There is a clear need for better therapeutics for both drug resistant and susceptible TB, particularly now to help reduce the increase in TB incidence and fatality arising from COVID-19.

There has been a recent push to develop host-directed therapies (HDTs), akin to immunotherapy which has been a breakthrough in cancer treatment. HDTs are expected to work against both drug susceptible and resistant TB, provide a treatment option that the pathogen is unlikely to develop resistance to, shorten treatment duration, boost the immune response, and ameliorate pathology associated with severe disease, and so could become an important tool in the fight against infectious diseases like TB [3]. Perhaps the most studied HDT is vitamin D, which boosts macrophage responses to control *M.tb* growth. However, clinical trials remain inconclusive as to whether Vitamin D helps control *M.tb* burden in people [4]. Initial studies aimed at supplementing the cytokine response (with IL-2 or IFNγ) have had limited success, perhaps because cytokines need to be applied early in infection for maximal effect. In contrast, corticosteroids and metformin have been used to reduce pathology during TB infection [3,5]. However, these are broad approaches and there are still no targeted HDTs in the clinic for TB.

Promising HDTs need to be active in both human macrophages (the primary niche for *M.tb*) and granulomas, which are the pathologic hallmark of TB. Granulomas are multicellular structures that contain bacteria, however it is hard for antibiotics to penetrate the granulomas and these structures provide a unique environment that causes some antibiotics to lose efficacy. These structures likely contribute to why TB therapy takes so long, and remain a challenge for development of new therapies. One approach for HDTs is to target cell death pathways which can impact the immune response. Historically, cell death has been mainly described as either apoptosis or necrosis. More recently, this has been expanded to include pyroptosis, necroptosis, and ferroptosis [6–8]. Apoptosis (Programmed Cell Death) has long been suggested as a mechanism to control TB, since avirulent or attenuated mycobacteria induce more apoptosis than virulent *M.tb,* some *M.tb* virulence factors have been identified that inhibit apoptosis, and induction of apoptosis leads to reduced *M.tb* growth in human and murine macrophages [6,7, 9]. Although this has been contested, recent work with apoptosis deficient mice has confirmed that apoptosis contributes to *M.tb* control in vivo [10], confirming that *M.tb* dampens apoptosis as a survival strategy. Apoptosis can be induced through two main pathways: the intrinsic or mitochondrial pathway and the extrinsic or death receptor-mediated pathway. The extrinsic pathway involves death receptors like Fas and tumor necrosis factor receptor (TNFR) and their ligands (Fas ligand, TNFα) and caspase-8 activation. The intrinsic or mitochondrial pathway is regulated by the BCL-2 protein family which consists of effectors BAK and BAX, anti-apoptotic BCL-2 proteins (BCL-2, MCL-1, BCL-X_L_, A1, BCL-W), and pro-apoptotic BH3-only proteins (BIM, PUMA, NOXA, BID, BAD, BMF, BIK). BAK and BAX oligomerize and form pores in the mitochondrial membrane, leading to cytochrome C release; and BAK and BAX activity is regulated by the anti-apoptotic and pro-apoptotic proteins [11]. The intrinsic and extrinsic pathways merge at caspase-3 activation, leading to DNA damage and apoptosis. A hallmark of apoptosis is that cells die with intact membranes, which limits inflammatory responses and efferocytosis of these apoptotic cells contributes to antigen presentation and T cell activation during *M.tb* infection. In contrast, necrosis is associated with increased *M.tb* growth and necrotic cells have membrane damage which releases pro-inflammatory mediators into the environment [6,7,12]. Thus, targeting apoptosis should aid in TB control on multiple levels: limiting *M.tb* growth in host macrophages and increasing antigen presentation, thereby boosting both the immune and adaptive response to TB while limiting damaging inflammation associated with disease.

Although inducing apoptosis has been a focus for boosting cancer treatment and polymorphisms in MCL-1 [13] and BCL-2 [14] are associated with susceptibility to TB disease, we were the first to interrogate inducing apoptosis through targeted inhibition of the anti-apoptotic BCL-2 proteins (specifically, MCL-1) for TB treatment [15]. Our previous work showed that targeted MCL-1 inhibition reduced *M.tb* growth in human macrophages, but that inhibition of multiple anti-apoptotic BCL-2 proteins (with pan inhibitors that inhibited anti-apoptotic MCL-1, BCL-2, and BCL-X_L_) was required to reduce *M.tb* growth in a more complex model of human granuloma-like structures [15]. We did not think the pan inhibitors were viable HDTs for TB however, since cancer clinical trials with the pan inhibitor ABT-263/navitoclax have been hampered due to toxicity concerns related to reduced platelet counts through inhibition of BCL-X_L_ [11]. Instead, we hypothesized that we could specifically target MCL-1 and BCL-2 (and not BCL-X_L_) to reduce *M.tb* growth in macrophages and the more complex granulomas. Since we are not inhibiting BCL-X_L_ we expect there to be minimal to no toxicity concerns associated with platelet loss. Intriguingly, there is only one manuscript to date that interrogated chemically inducing extrinsic apoptosis in mice and found this significantly limited *M.tb* growth in vivo [10]. No one has investigated inducing intrinsic apoptosis as a way to limit *M.tb* infection.

Here we interrogated if specific inhibition of MCL-1 and BCL-2, using combinations of these inhibitors that are efficacious in mouse cancer models and have advanced to clinical trials or are FDA-approved for cancer therapy [11,16–19] would: 1) limit *M.tb* burden in human macrophages more than inhibition of just one target, 2) limit growth of both drug susceptible and resistant *M.tb* stains, 3) further reduce *M.tb* burden when combined with front line antibiotics INH or RIF, and 4) limit *M.tb* growth in a pre-clinical human in vitro granuloma model, which we previously developed [20]. In this model, the in vitro granuloma-like structures contain the predominant cells within human in vivo granulomas. The structures are up to 15 cell layers thick, and this model is used to study infectious and non-infectious granulomatous diseases, including *M.tb* and sarcoidosis, respectively [20–23]. This model recapitulates several signatures observed in sarcoidosis, like up-regulation of Th1 immune response and alternatively activated M2-like macrophages [23,24]. Thus, this multicellular granuloma model provides a more readily tractable, accessible, and affordable throughput bridge model for assessing HDT and antibiotic efficacy in complex granulomas than animals.

## 2. Material and methods

### 2.1 Study approval

Peripheral blood mononuclear cells (PBMCs) were isolated from human peripheral blood collected from healthy donors, following Texas Biomed approved Institutional Review Board (IRB) protocols. All donors for these studies provided informed, written consent. Texas Biomed Institutional laboratory Animal Care and Use Committee (IACUC) approved all protocols involving animal samples.

### 2.2. Isolation and culture of human monocyte-derived macrophages (MDMs)

MDMs were prepared as described elsewhere [25,26]. Briefly, heparinized blood was layered on a Ficoll-Paque cushion (GE Healthcare, Uppsala, Sweden) to allow for collection of PBMCs. PBMCs were cultured in RPMI 1640 (Life Technologies, Carlsbad, CA) with 20% autologous serum in Teflon wells (Savillex, Eden Prairie, MN) for 5 days at 37 ◦C/5% CO_2_. MDMs were harvested and adhered to tissue culture dishes for 2–3 h in RPMI with 10% autologous serum, lymphocytes were washed away, and MDMs were incubated overnight in RPMI with 10% autologous serum. Such MDM monolayers are 99% pure and viable.

### 2.3. Isolation and culture of murine bone marrow-derived macrophages (BMDMs)

8–9-week-old female BALB/c and C57BL/6 mice (Jackson Laboratory, Bar Harbor, ME), were euthanized by CO_2_ asphyxiation. For BAX/BAK KO, Rosa-CreERT2 Bax^f/f^ Bak^−/−^ mice were treated with tamoxifen for five days to induce deletion of BAX, then mice were euthanized and tibias and femurs collected [27]. BMDMs were obtained from tibias and femurs as previously described [28]. BMDMs were frozen in DMEM supplemented with 30% L-cell conditioned media, 20% heat inactivated-FBS (HI-FBS), 10 units penicillin/streptomycin (Pen/Strep), 50.3 μM β-mercaptoethanol and 10% dimethylsulfoxide. L-cell conditioned media was obtained by collecting media from confluent NCTC clone 929 (ATCC # CCL-1) cells. The day before the experiment, BMDMs were thawed and plated in tissue culture dishes in DMEM supplemented with 10 units Pen/Strep and 10% HI-FBS. The day of the experiment, cells were washed twice with DMEM to remove the antibiotics from the culture medium.

### 2.4. Bacterial strains

Lyophilized *M.tb* H_37_R_v_ (27294) was obtained from the American Tissue Culture Collection (ATCC, Manassas, VA). *M.tb* H_37_R_v_ lux was created and used as described [29]. This bacterial strain contains the entire bacterial Lux operon cloned in a mycobacterial integrative expression vector. The previously validated *M.tb* H_37_R_v_ *InhA* T(−8) INH R strain was obtained from Dr. Sanjay Jain, Johns Hopkins University School of Medicine, Baltimore, MD [30]. *M.tb* strain HN563 (NR-18986), a clinical isolate with resistance to RIF and INH, was obtained through BEI resources, NIAID, NIH. The clinical strain of *M.tb* resistant to INH and EMB was obtained from the OSU Clinical Microbiology Laboratory [29]. Single cell suspensions of bacteria were prepared as previously described [31,32]. The bacteria concentration and degree of clumping (<10%) were determined with a Petroff-Hausser Chamber. This method results in ≥90% viable bacteria, as determined by colony forming unit (CFU) assay.

### 2.5. M.tb infection of macrophages

Single cell suspensions of *M.tb* in RHH [for MDM infections: 10 mM HEPES (Life Technologies) and 0.1% human serum albumin (CSL Behring, King of Prussia, PA) in RPMI] or DHH [for BMDM infections: 10 mM HEPES and 0.1% human serum albumin in DMEM] were added to the macrophages at MOI 1 and cells were incubated for 2 h at 37 ◦C, with the first 30 min on a platform shaker. Macrophages were then washed and incubated in RPMI with 2% autologous serum (for MDMs) or DMEM with 2% HI-FBS (for BMDMs) for the indicated times. Where indicated, the MCL-1 and BCL-2 inhibitors, antibiotics, or solvent control (dimethyl sulfoxide, DMSO), were added after this wash step. The MCL-1 inhibitors S63845, MIK665, and AZD5991, and BCL-2 inhibitor ABT-199 were purchased from Selleckchem (Houston, TX), and the MCL-1 inhibitor AMG 176 was from MedChemExpress (Monmouth Junction, NJ). The antibiotics RIF and INH were purchased from Sigma (St. Louis, MO). All inhibitors and antibiotics were maintained throughout the course of infection. Cells were observed with an EVOS XL Core Imaging System to ensure monolayer integrity was maintained throughout the course of all experiments (Figs S1 and S3).

### 2.6. Culture, infection, staining and imaging of in vitro human granulomas

*In vitro* TB granulomas were generated as described elsewhere [15, 20,21]. Briefly, human peripheral blood was collected from healthy Mantoux tuberculin skin test (TST) and/or IFNγ release assay (IGRA)--positive individuals. PBMCs were isolated as above, and were immediately infected with single cell suspensions of *M.tb* at MOI 1 in RPMI with 10% autologous serum, then incubated at 37 ◦C/5% CO_2_. Serum was replenished after 4 days. With this method, cells typically start to aggregate around day 4 and the granulomas are stable for up to 12 days. For CD56 staining, cells on glass coverslips were fixed with 4% paraformaldehyde (PFA) for 20 min and then washed twice with PBS and incubated in blocking buffer (5% BSA/10% HI-FBS/PBS) overnight at 4 ◦C. Cells were then incubated with 488-tagged antibodies against CD56 (clone HCD56, cat # 318311, lot B249761, Biolegend), for 1 h in blocking buffer, washed, stained with DAPI and mounted with ProLong Gold antifade reagent. Cells were imaged with a Zeiss LSM 800 confocal microscope. Live cell imaging was performed using the Cytation 5 microscope paired with BioSpa (Agilent, Santa Clara, CA) to maintain cells at 37 ◦C/5% CO_2_ for the duration of the movie. Images were acquired every 6 h (0–138 h time period) with a 4x objective. See [20] for details regarding CD11b, CD3 and *M.tb* staining in granulomas. For trypan blue staining, equal volume of 0.4% trypan blue was added to structures at days 4 or 7 post infection. Cells were immediately centrifuged at 100 g for 5 min (to ensure granuloma structures remained attached to the tissue culture wells), washed with PBS and then immediately imaged with a 20x objective on an EVOS XL Core Imaging System. As a positive control, structures were treated with 0.1% Triton-X 100 10 min prior to trypan blue staining, this resulted in disruption of granuloma structures and > 99% cells becoming trypan blue-positive. For CFU experiments antibiotics, MCL-1 and BCL-2 inhibitors, or solvent controls were added as described in the figure legends. The antibiotic moxifloxacin (MX) was purchased from Selleckchem, and linezolid (LNZ) and cycloserine (CS) were purchased from Sigma.

### 2.7. M.tb growth assays

#### 2.7.1. Intracellular growth

Intracellular growth was assayed with two approaches. For CFU assays, infected macrophages and granulomas were lysed as described previously [20,33]. Lysates were diluted, and plated on 7H11 agar (Remel, San Diego, CA). The number of CFUs was enumerated after growth for at least 3 weeks at 37 ◦C. For luciferase growth assays, macrophages were infected with *M.tb*-lux, and bacterial bioluminescence was measured in relative luminescence units (RLUs) every 24 h for up to 7 days with a GloMax Microplate Reader (Promega, Madison, WI) [29].

#### 2.7.2. Broth cultures

*M.tb* was cultured in 7H9 broth (BD, Franklin Lakes, NJ) with S63845 + ABT-199 for 4 days, followed by dilutions in 7H9 and plating on 7H11 agar. The number of CFUs was enumerated after growth for at least 3 weeks at 37 ◦C.

### 2.8. Cell death assays

Macrophages in 96 well plates were infected with *M.tb* at MOI 1 and caspase activity and cell death was assayed in triplicate wells with Caspase-3/7 or CellTiter Glo Assays (Promega), respectively, following the manufacturer’s instructions.

### 2.9. Western blotting

Cells were washed with PBS, then lysed with TN1 lysis buffer (125 mM NaCl, 50 mM Tris, 10 mM EDTA, 1% Triton X-100, 10 mM Na_4_PO_7_, 10 mM NaF with 10 mM Na_3_VO_4_, 10 μg/ml aprotinin, and 10 μg/ml leupeptin) at 4 ◦C. Lysates were centrifuged (10,000 g, 4 ◦C, 10 min) to remove cell debris, then a Pierce BCA assay (Thermo Scientific, Waltham, MA) was performed to determine protein concentration. Equivalent amounts of denatured and reduced protein were separated by SDS-PAGE and analyzed by Western blot using antibodies against caspse-3 (cat# 9662, lot 19), poly ADP-ribose polymerase (PARP; clone 46D11, cat# 9532, lot 10), and β-actin (clone 13E5, cat# 5125, lot 7), obtained from Cell Signaling (Danvers, MA). The membranes were developed using clarity ECL reagent on a UVP ChemStudio 815 system (Analytik Jena US LLC, Upland CA).

### 2.10. Statistics

A minimum of at least three independent experiments was performed, with cells from at least three different human donors, unless indicated otherwise. Although the trend was the same for each donor, the magnitude of change differed among donors. Consequently, results from each experiment were normalized to an internal control and an unpaired one-tailed Student’s *t*-test (if comparing two groups) or ANOVA (when comparing more than 2 groups) were performed on the normalized data, with *P* < 0.05 considered significant.

## 3. Results

### 3.1. Combination inhibitors against MCL-1 and BCL-2 reduce M.tb growth in human and murine macrophages

**Fig. 1.**
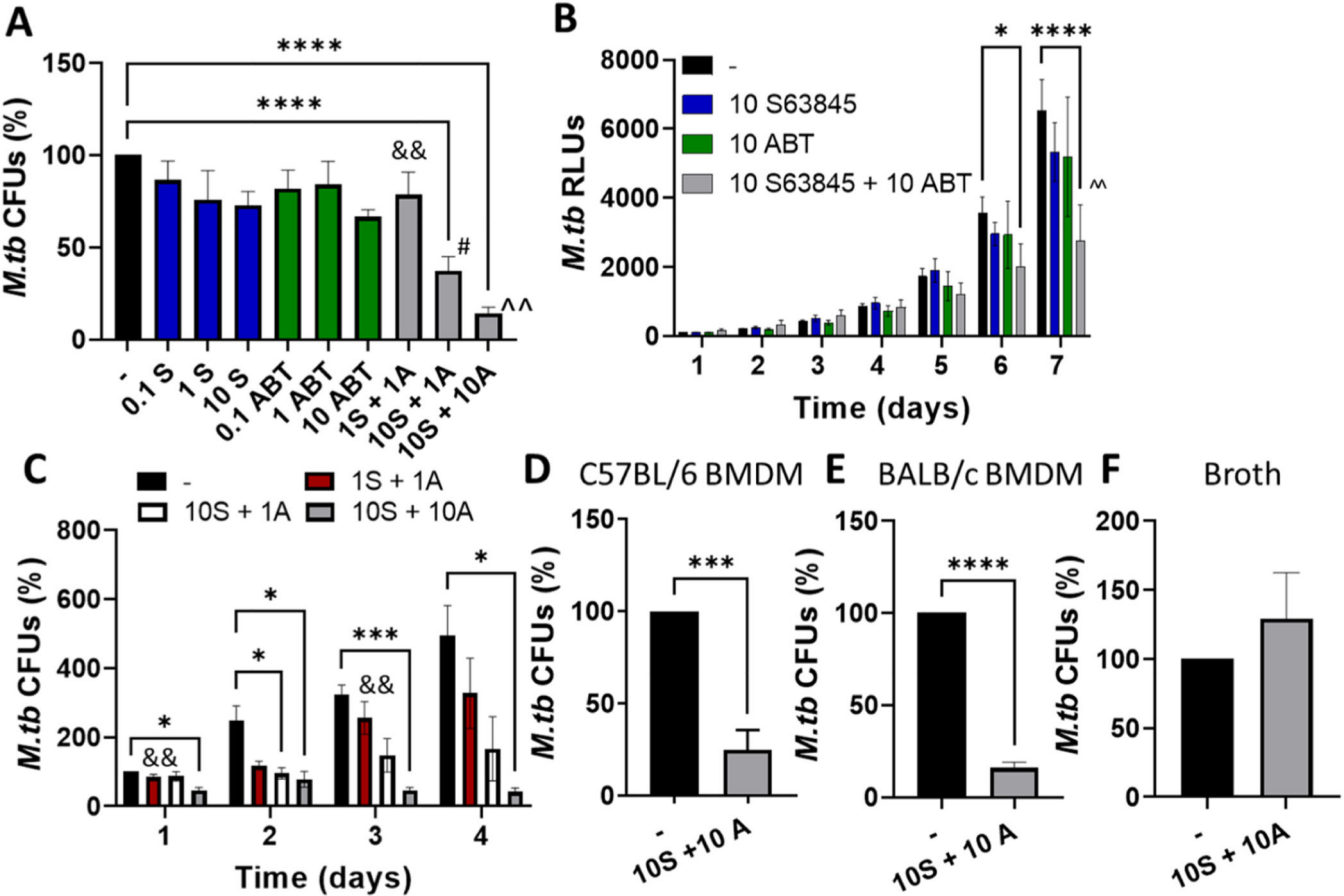
MCL-1 and BCL-2 combination therapy limits *M.tb* growth in human and murine macrophages. A-C) MDMs were infected with *M.tb* (A, C) or *M.tb*-lux (B), then treated with the MCL-1 inhibitor S63845 (S) and BCL-2 inhibitor ABT-199 (A), alone or in combination, at the indicated concentrations in μM. A) After 4 days, cells were lysed and CFUs enumerated. Results are % *M.tb* CFUs, relative to the untreated control and are the mean ± SEM of at least three independent experiments. B) *M.tb* growth was enumerated by luciferase activity over time. Results are relative luciferase units (RLUs), normalized to untreated RLUs at day 1 and are mean ± SEM of at least five independent experiments. C) *M.tb* growth was enumerated by CFU over time. Results are relative to untreated CFUs at day 1 and are mean ± SEM of at least four independent experiments. D,E) BMDMs from C57BL/6 (D) and BALB/c (E) mice were infected with *M.tb*, then treated with 10 μM S63845 + 10 μM ABT-199. After 4 days cells were lysed and CFUs enumerated. Results are % *M.tb* CFUs, relative to the untreated control. Results are mean ± SEM of at least three independent experiments. F) *M.tb* was cultured in 7H9 broth with 10 μM S63845 + 10 μM ABT-199. After 4 days CFUs were enumerated. Results are % *M.tb* CFUs, relative to the untreated control and are the mean ± SEM of three independent experiments. A-F) * indicates a significant difference from the untreated control, * p < 0.05, * ** p < 0.001, * ** * p < 0.0001; ^^ indicates that 10 S+ 10 A was significantly different from the same concentration of either inhibitor alone, p < 0.01; # indicates that 10 S+ 1 A was significantly different from 1 A alone, p < 0.05; and && indicates that 1 S+ 1 A was significantly different from 10 S+ 10 A, p < 0.01.

We have previously shown that MCL-1 inhibitors reduce *M.tb* growth in human macrophages, but that inhibition of multiple proteins in the MCL-1/BCL-2 family was more effective and required to reduce *M.tb* growth in more complex human in vitro granuloma structures [15]. Since this work, newer more potent MCL-1 inhibitors have been reported, including ones with efficacy in mouse cancer models. We were interested in determining if a new MCL-1 inhibitor, in combination with a BCL-2 inhibitor, would limit *M.tb* growth to a similar extent as the pan BCL-2 family inhibitors. To assess this, we infected human monocyte-derived macrophages (MDMs) with *M.tb*, then treated with MCL-1 or BCL-2 inhibitors alone and together. We tested the MCL-1 inhibitor S63845 and the BCL-2 inhibitor ABT-199 at concentrations similar to those used in the literature that specifically induce apoptosis in a BAX/BAK and/or caspase-dependent manner [19,34–37]. Of note, 10 μM S63845 alone reduced *M.tb* burden in macrophages by 27.25 ± 7.47% (Fig. 1A), indicating this is more potent than the previous MCL-1 inhibitors, which required a higher concentration to reduce *M.tb* growth [15]. 10 μM ABT-199 alone reduced *M.tb* burden by 33.49 ± 3.81%, to a similar extent as the MCL-1 inhibitor S63845. In contrast to single inhibitors, combination therapy was significantly more potent at reducing *M.tb* growth in macrophages, and this occurred in a concentration dependent manner (Fig. 1A). At 10 μM S63845 + 1 μM ABT-199, intracellular growth was reduced by 62.77 ± 7.74%, N = 10, and at 10 μM S63845 ± 10 μM ABT-199, intracellular growth was reduced by 85.76 ± 3.27%, N 9. This marked level of growth reduction is particularly notable with use of primary human macrophages.

We next determined the kinetics of *M.tb* growth inhibition using a luciferase-expressing *M.tb* strain, focusing on 10 μM of S63845 and/or ABT-199. Although this proved to be less sensitive than CFUs at detecting differences in *M.tb* growth, we observed that combination therapy was significantly more potent than either inhibitor alone, and that reduction in *M.tb* growth was maintained for at least seven days post infection (Fig. 1B). Using the more sensitive CFU assay, we repeated these kinetic experiments, but focused early during the first four days of infection. After just one day of treatment, we could detect a significant reduction in *M.tb* growth by 53.88 ± 7.90% with 10 μM of S63845 and ABT-199 (Fig. 1C). This level of control was maintained throughout the four days, at day four *M.tb* growth was reduced by 57.92 ± 10.94% relative to day one, whereas *M.tb* growth increased in the untreated group. Combinations of lower concentrations of the inhibitors also reduced *M.tb* growth. We confirmed that MDMs remained adherent and the monolayer was intact throughout the course of the experiments, but subtle morphological changes (cells shrinking and rounding) characteristic of apoptosis did occur. We observed morphological changes in a concentration and time dependent manner, and this was noticeable with 10 μM of S63845 and ABT-199 already at day 1 (Fig S1, [7]).

S63845 binds human MCL-1 with 6-fold higher affinity than murine MCL-1, yet was still able to reduce tumor burden in mice [19]. We wanted to determine if the inhibitors would reduce *M.tb* growth in murine macrophages. We treated *M.tb-*infected murine bone marrow-derived macrophages (BMDMs) with 10 μM of S63845 + 10 μM ABT-199 and assessed *M.tb* growth by CFUs. Similar to human macrophages, S63845 + ABT-199 significantly reduced *M.tb* burden in murine BMDMs, from both C57BL/6 and BALB/c mice, by 75.18 ± 10.72% (N =4) and 84.10 ± 3.10% (N = 3) respectively, showing these inhibitors are also potent in murine macrophages and this is not strain dependent (Fig. 1D,E).

We next determined whether the MCL-1 + BCL-2 inhibitors had a direct impact on *M.tb* growth. As expected, 10 μM of S63845 ± 10 μM ABT-199, which significantly reduced *M.tb* growth in human and murine macrophages (Fig. 1A-E) did not alter *M.tb* growth in broth culture (Fig. 1F). This dichotomous activity is expected for HDTs that target host molecules to improve host immune responses (15).

### 3.2. Combination inhibitors against MCL-1 and BCL-2 induce macrophage apoptosis

**Fig. 2.**
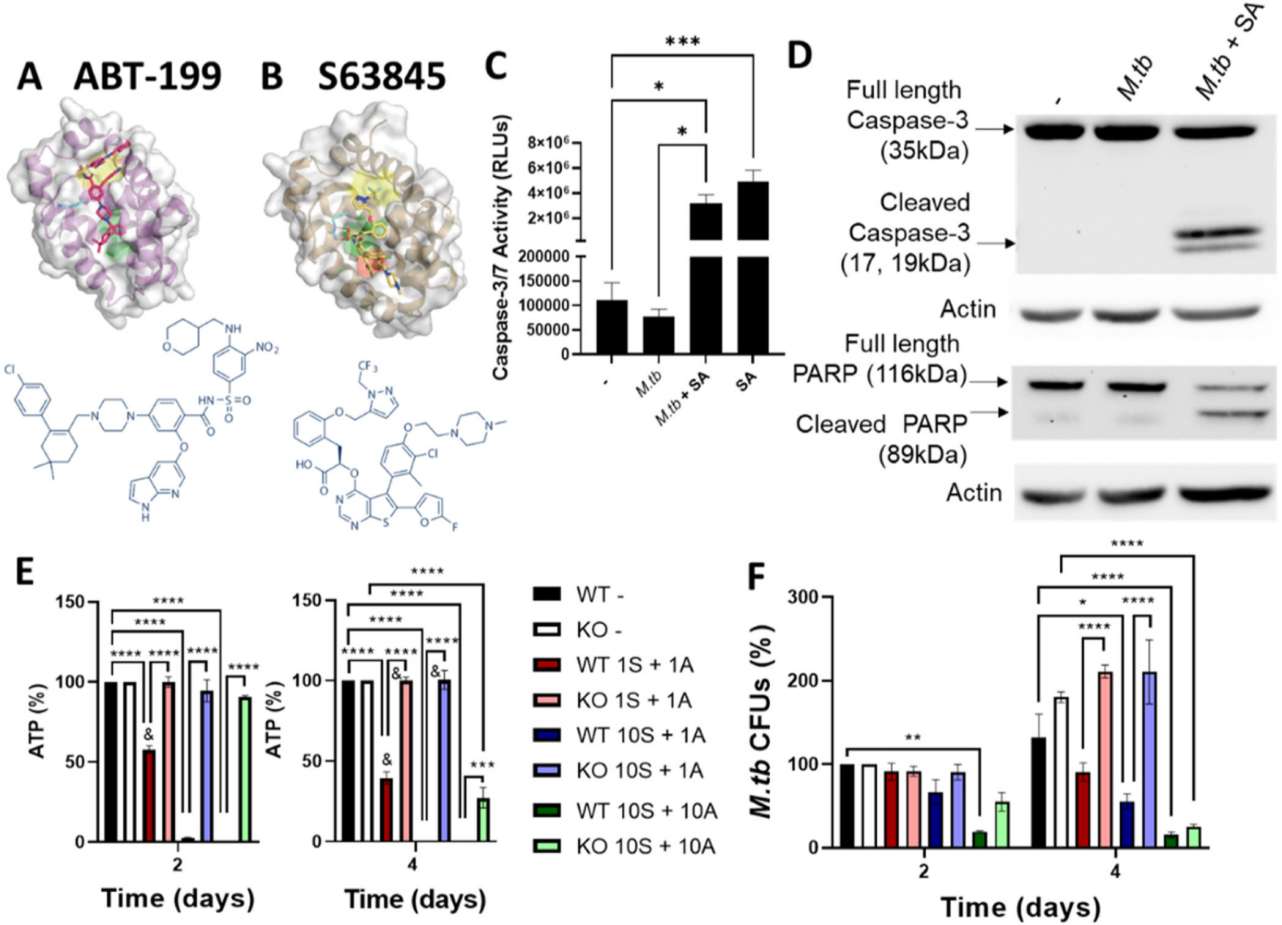
MCL-1 and BCL-2 combination therapy induces apoptosis in *M.tb* infected human macrophages. A and B) Chemical structures of ABT-199 and S63845, alone and in complex with BCL-2 and MCL-1, respectively. C and D) MDMs were infected with *M.tb*, then treated with 10 μM MCL-1 inhibitor S63845 + 10 μM BCL-2 inhibitor ABT-199. After 4 h, caspase activity was enumerated with the Caspase-3/7 Assay (C) or protein lysates were collected for Western blotting (D). C) Results are mean ± SEM of three independent experiments, * p < 0.05, * ** p < 0.001. D) Representative blot of three independent experiments, see Fig S2 for un-cropped blots. E and F) BMDMs from BAX+BAK KO or control (WT) mice were infected with *M.tb*, then treated with S63845 + ABT-199 at the indicated concentrations (μM). After 2 or 4 days cells were lysed and cell death assessed by CellTiter Glo Assay (E) and CFUs enumerated (F). E) Results are % macrophage ATP (E) or *M.tb* CFUs (F), relative to the untreated control. Results are mean ± SEM of three experiments. * indicates a significant difference between the indicated groups, * p < 0.05, * * p < 0.01, * ** p < 0.001, * ** * p < 0.0001; & indicates that 10 S+ 10 A was significantly different from the indicated group, p < 0.01. Chemical structures were obtained from the manufacturer’s website (Selleckchem, https://www.selleckchem.com, accessed 2/16/23), Structures complexed with BCL-2 and MCL-1 are reproduced from [11].

ABT-199, which is highly specific to BCL-2, binds BCL-2 with subnanomolar affinity (K_i_ < 0.010 nM) and disrupts BCL-2 binding to the pro-apoptotic BIM, which likely frees BIM to bind and activate BAX/BAK and induce apoptosis (Fig. 2A) [11,38]. S63845, which is specific to MCL-1, binds MCL-1 with nanomolar affinity (K_i_ < 1.2 nM) and inhibits binding of anti-apoptotic MCL-1 to BAX/BAK, thus inducing apoptosis (Fig. 2B) [11,19]. Both S63845 and ABT-199 induce intrinsic apoptosis, which is initiated by BAX/BAK oligomerization at the mitochondrial membrane, leading to cytochrome C release, caspase-3 and caspase-7 activation, and PARP cleavage, DNA damage, and apoptosis [12,16–18].

As reported for cancer cell lines using either inhibitor alone or in combination [19,35,38], we confirmed that the MCL-1 inhibitor S63845 and the BCL-2 inhibitor ABT-199 induced apoptosis in *M.tb-*infected MDMs, by assessing key hallmarks of apoptosis including caspase-3 and caspase-7 activity (Fig. 2C) and PARP and caspase-3 cleavage (Fig. 2D). S63845 and ABT-199 induced apoptosis in both uninfected and infected macrophages (Fig. 2C). We note that although these inhibitors induced apoptosis (and so predictably would ultimately impair macrophage viability), at the concentrations and time points tested, the cell monolayers remained intact (Fig S1). This suggests that early apoptotic signaling events, and not massive cell disruption, controls *M.tb* growth. Indeed, cell death can be beneficial and detrimental to pathogens, depending on the cell death modality (reviewed in [39]). Increased necrosis and ferroptosis, two distinct types of inflammatory cell death, are associated with increased *M.tb* growth and dissemination [8,40]. In contrast, apoptosis is linked to improved control of *M.tb* within macrophages [6,10,15,39–41] through unclear mechanisms, although potentially due to caspase activity [39].

S63845 and ABT-199 mediated induction of apoptosis is BAX/BAK and caspase dependent, confirming that these inhibitors specifically induce apoptosis through on-target and specific actions [19,38]. We had previously confirmed that the ability of MCL-1 inhibitors to control *M.tb* growth requires caspase activity, and so is specific to apoptosis induction [15]. To verify that the combination of S63845 and ABT-199 controls *M.tb* growth due to on-target induction of apoptosis, we obtained BMDMs from mice that were knocked out for both BAX and BAK, the two main apoptosis effectors [27]. As expected, these BMDMs were significantly more resistant to S63845 and ABT-199 mediated cell death than the control BMDMs (Fig. 2E), proving that S63845 and ABT-199 induced apoptosis in a BAX/BAK-dependent manner in murine macrophages, as reported for other cell types [19,38]. Similar to our observations in MDMs (Fig S1), the BMDM monolayers remained intact throughout the experiment. However, induction of apoptosis corresponded with subtle phenotypic changes of the WT BMDMs, with cells rounding up in a concentration- and time-dependent manner (Fig S3). Congruent with the cell death findings, we noted that S63845 and ABT-199 mediated control of *M.tb* growth was dose-, time-, and BAX/BAK-dependent in BMDMs (Fig. 2F), and the ability of S63845 and ABT-199 to control *M.tb* was only observed when they also induced cell death (Fig. 2E&F). Intriguingly, 10 μM S63845 + 10 μM ABT-199 induced cell death, phenotypic changes, and reduced *M.tb* growth after 4 days of treatment, even in BAX/BAK KO BMDMs, although to a significantly lesser extent than in WT BMDMs. We suspect that this is due to the expression of BOK, an additional apoptosis effector that is expressed in murine BMDMs, and able to induce apoptosis even in the absence of BAX and BAK (reviewed in [42]). However, it is also possible that in murine macrophages, 10 μM S63845 + 10 μM ABT-199 controlled *M.tb* growth through apoptosis-independent functions. For this reason we used 10 μM S63845 + 1 or 10 μM ABT-199 throughout the rest of the studies, and we note that the ability of 10 μM S63845 + 1 μM ABT-199 to significantly reduce *M.tb* growth was completely abrogated in the BAX/BAK KO BMDMs.

We also observed increased *M.tb* growth in BAX/BAK KO relative to control BMDMs, similar to what has been reported in vivo in mice [10], likely due to baseline levels of apoptosis during infection that moderately limit *M.tb* growth. We also noted that S63845 and ABT-199 were less effective at controlling *M.tb* growth in BMDMs (Fig. 2F) when compared to human MDMs (Fig. 1C), with 10 μM S63845 + 1 μM ABT-199 reducing *M.tb* growth by 61.7% in MDMs but only 33.4% in BMDMs at day 2, relative to the untreated. We posit that this is due to reduced binding of S63845 to murine MCL-1 [19], although after 4 days we observed similar levels of *M.tb* control in MDMs (66.3%) and BMDMs (58.0%). Altogether, these results support that S63845 and ABT-199 mediated control of *M.tb* growth is through induction of apoptosis.

### 3.3. Combination inhibitors against MCL-1 and BCL-2 reduce growth of drug resistant M.tb in macrophages

**Fig. 3.**
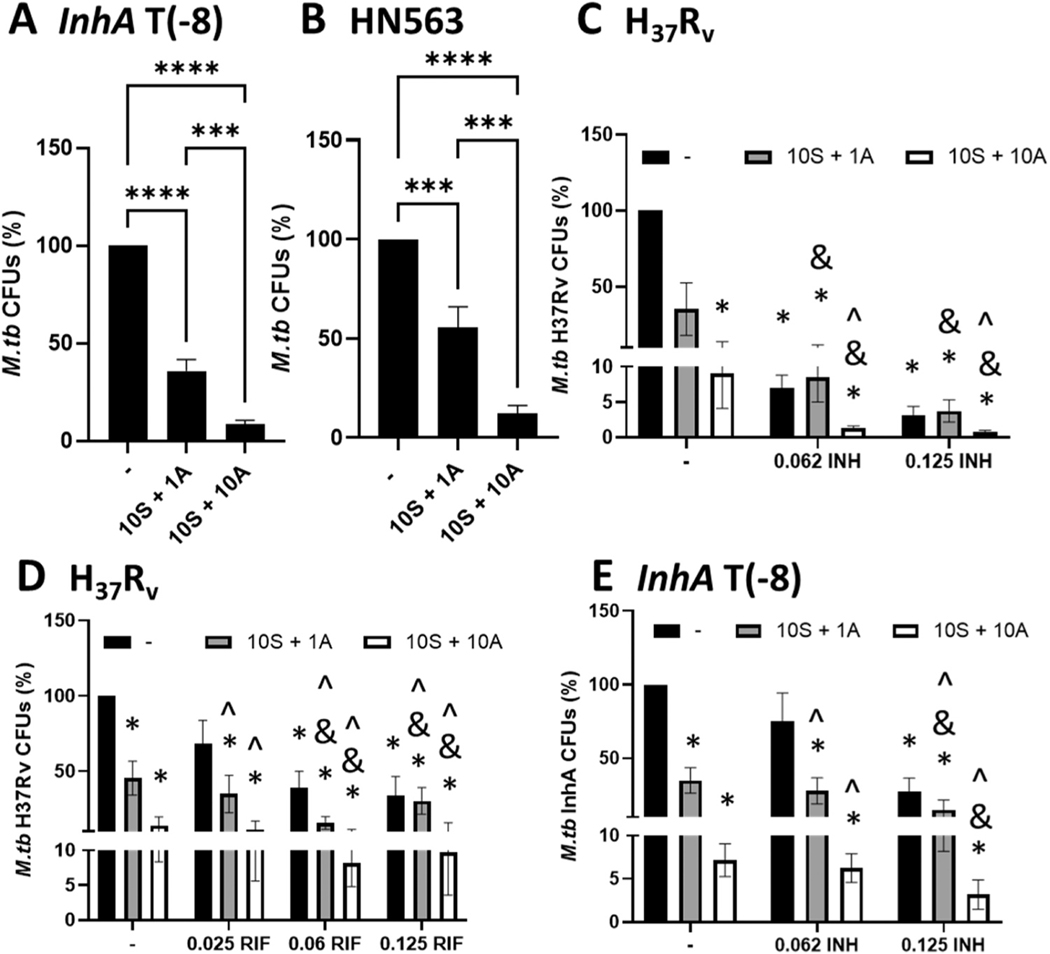
MCL-1 and BCL-2 combination therapy limits drug resistant *M.tb* growth in human macrophages. MDMs were infected with drug resistant *M.tb*: InhA T(−8) (A,E: INH R) or HN563 (B: RIF + INH R) or drug susceptible *M.tb* (C,D: H_37_R_v_), then treated with the indicated concentrations (μM) of S63845 + ABT-199 (S+A) without or with the indicated concentration (μg/ml) of INH or RIF. After 4 days, macrophages were lysed and CFUs enumerated. Results are % *M.tb* CFUs, relative to the untreated control. A-B) Results are the mean ± SEM of at least five independent experiments, * ** p < 0.001, * ** * p < 0.0001. C-E) Results are the mean ± SEM of at least three independent experiments, * indicates a significant difference from untreated control, p < 0.05; & indicates a significant difference between S+A and S+A + INH/RIF, p < 0.05; ^ indicates a significant difference between INH/RIF and INH/RIF + S+A, p < 0.05.

Since MCL-1 + BCL-2 inhibitors are HDTs that enable macrophages to better limit *M.tb* growth, we expected that MCL-1 and BCL-2 inhibitors that induce macrophage apoptosis would enable the macrophage to control intracellular growth of any *M.tb* strain, including drug susceptible and resistant *M.tb*. To test this, we infected MDMs with a previously validated and published *M.tb* strain with a point mutation in the InhA promoter region that mediates resistance to the front-line drug INH [*InhA* T(−8) [30]] and a clinical MDR strain (HN563) that BEI reports is resistant to INH and RIF, and recent studies have also described as being pyrazinamide resistant [43,44]. We chose these two strains since INH and RIF are the two most common antibiotic resistance profiles observed in the clinic for TB [1,45,46]. As expected, S63845 + ABT-199 markedly reduced drug resistant *M.tb* growth in macrophages in a concentration dependent manner (Fig. 3A,B) to a similar extent as the drug susceptible *M.tb* strain H_37_R_v_ (Fig. 1). 10 μM S63845 + 1 μM ABT-199 reduced *M.tb InhA* T(−8) growth by 64.16 ± 5.81%, and 10 μM S63845 + 10 μM ABT-199 reduced *M.tb InhA* T −8) growth by 91.49 ± 2.00%, N = 5. 10 μM S63845 + 1 μM ABT-199, and 10 μM S63845 ± 10 μM ABT-199 reduced *M.tb* HN563 growth by 44.47 ± 10.51 and 87.56 ± 3.91%, respectively, N = 5. As noted earlier, this level of growth reduction is rarely seen when using primary human macrophages, particularly with drug resistant *M.tb*.

We next wondered if S63845 + ABT-199, in conjunction with antibiotics, would further reduce intra-macrophage *M.tb* growth more than antibiotics or S63845 + ABT-199 alone. To this end, we treated *M.tb-*infected macrophages with 10 μM S63845 + 1 or 10 μM ABT-199. We also tested a range of INH and RIF concentrations, using 1x minimal inhibitory concentration (MIC) [47] and lower. For the drug susceptible *M.tb* strain H_37_R_v_, we found that 0.062 and 0.125 μg/ml INH [0.5x and 1x published MIC, respectively [47]] significantly reduced *M.tb* growth in MDMs (Fig. 3C). Excitingly, the addition of 10 μM S63845 + 10 μM ABT-199 to INH was significantly more potent in reducing *M.tb* growth than either 10 μM S63845 + 10 μM ABT-199 (90.95 ± 4.96% growth inhibition) or INH alone (0.062 μg/ml INH reduced growth by 93.04 ± 1.85%), and 10 μM of S63845 + 10 μM of ABT-199 + 0.062 μg/ml INH reduced *M.tb* growth by 98.70 ± 0.34%. This was also observed when using a slightly higher dose of INH, 10 μM of S63845 + 10 μM of ABT-199 + 1.25 μg/ml INH reduced *M.tb* growth by a striking 99.19 ± 0.20% (Fig. 3C). We observed a similar trend when combining S63845 + ABT-199 with RIF, that addition of S63845 + ABT-199 to RIF was significantly more potent than RIF or S63845 + ABT-199 alone (Fig. 3D). Addition of 10 μM S63845 + 10 μM ABT-199 to 0.125 μg/ml RIF (1x MIC [47]) led to 90.25 ± 6.17% reduction of *M.tb* growth, while 0.125 μg/ml RIF alone reduced growth by 66.35 ± 12.74% or 10 μM S63845 + 10 μM ABT-199 reduced growth by 85.94 ± 5.71% (Fig. 3D). These results indicate that addition of S63845 ABT-199 could potentiate current antibiotic therapy to improve outcomes.

We also determined if addition of S63845 + ABT-199 could potentiate treatment for drug resistant *M.tb*. We focused on INH resistant *M.tb*, since this is the most prevalent drug resistance in the clinic and a key front line drug [46]. In contrast to the drug susceptible *M.tb* strain H_37_R_v_, growth of the INH resistant *M.tb* strain was not significantly inhibited by 0.062 μg/ml INH in MDMs (Fig. 3E) and was inhibited by only 72.49 ± 8.99% at the higher concentration of INH (0.125 μg/ml) compared to a growth inhibition of 96.93 ± 1.32% for H_37_R_v_ at the same INH concentration (Fig. 3C,E). However, similar to *M.tb* strain H_37_R_v_, the addition of S63845 + ABT-199 to INH was significantly more potent than either S63845 + ABT-199 or INH alone at reducing INH R *M.tb* growth; 10 μM of S63845 + 1 μM of ABT-199 reduced *M.tb* growth by 64.16 ± 5.81% and 0.125 μg/ml INH reduced *M.tb* growth by 72.49 ± 8.99%, while 10 μM S63845 + 1 μM ABT-199 + 0.125 μg/ml INH reduced *M.tb* growth by 85.1 ± 6.72%; 10 μM of S63845 + 10 μM of ABT-199 reduced *M.tb* growth by 92.86 ± 1.90% and 10 μM S63845 + 10 μM ABT-199 0.125 μg/ml INH reduced *M.tb* growth by 96.83 ± 1.70% (Fig. 3E), indicating that addition of S63845 + ABT-199 could potentiate current antibiotic therapy to improve outcomes, even for drug resistant *M.tb*. In summary, S63845 + ABT-199 significantly reduced growth of drug susceptible and resistant *M.tb* in macrophages and combinations of S63845 + ABT-199 with INH or RIF led to a greater improvement in *M.tb* control than antibiotic alone.

### 3.4. Use of the human in vitro granuloma model for assessing ant-TB drug efficacy

Granulomas, which contain macrophages, multinucleated giant cells, lymphocytes, and fibroblasts are a hallmark of TB and these dense multicellular structures provide a niche for *M.tb* that is recalcitrant to antibiotics [22,48,49]. A stumbling block for development of anti-*M.tb* drugs has been a lack of readily accessible and cost effective animal models that reliably mimic human TB granulomas, and predict drug efficacy and safety in humans. For example, moxifloxacin (MX) and linezolid (LNZ) limit *M.tb* growth in murine models, but do not exhibit promising clinical efficacy at non-toxic concentrations in people [50]. In contrast, cycloserine (CS) does not effectively reduce *M.tb* burden in mice, but limits drug-resistant *M.tb* in patients [47,50]. This is likely due to the inability of some antibiotics to penetrate TB granulomas, and/or reduced activity in the human granuloma environment; which cannot be reliably assessed in murine models that exhibit different granuloma architecture relative to man [22,48,49].

We developed a human in vitro granuloma model to serve as a drug screening tool to identify promising compounds active in the unique TB granuloma [15,20,21]. This model involves adding *M.tb* to human PBMCs. In the absence of infection, PBMCs remain as single cell monolayers but upon infection with live *M.tb*, PBMCs form granuloma-like structures that are up to 15 cell layers thick, contain macrophages, multinucleated giant cells, CD4 + and CD8 + T cells, B cells, NK cells, and intracellular *M.tb* (Fig S4A-D, Movies S1,S2)[20,21]. Bacteria are seen exclusively within the granuloma-like structures rather than in adjacent areas and the in vitro granuloma structures that form exhibit a range of sizes (Fig S4A, C, Movie S1), similar to NHP granulomas in the lung [51]. Granulomas generated with cells from latent TB infected (LTBI), in comparison to naïve, individuals generate a significantly different response, showing a 2-fold increase in cellular aggregation (up to 15 cell layers thick in LTBI donors), significantly enhanced *M.tb* control, cell proliferation, production of pro-inflammatory cytokines, and altered location of lipid bodies in both granulomas and *M.tb* [20]. We also found that *M.tb* responses were altered in granulomas from LTBI and naïve donors [20]. This indicates that human infection status impacts early events in granuloma formation and function, and *M.tb* responses. Due to the impact that host infection status has on the response of these in vitro granuloma-like structures, in the current studies we generated in vitro granuloma like structures with PBMCs from LTBI donors since this better reflects the host immune status of individuals who are treated for TB.

To assess the utility of the granuloma model to serve as a drug screening tool that is predictive of efficacy in the clinic to treat TB, we tested the first line drug INH (which penetrates TB granulomas in people; [48]) and three drugs used for MDR TB: CS which has been effective in clinical trials but is associated with psychiatric adverse events, MX which does not enter the granuloma caseum [48], and LNZ which is associated with toxicity concerns at clinically relevant concentrations [50]. These drugs were also selected since CS is active in patients, but not mice, and MX and LNZ are active in murine studies but do not exhibit promising clinical efficacy; thus allowing us to determine if the human in vitro granuloma model better predicts drug efficacy in patients than a standard mouse model. Since INH and CS are potent at treating human TB, we expected them to control *M.tb* growth in in vitro granulomas. However, since MX does not effectively penetrate the granuloma structure and LNZ limits human TB only at high concentrations, we would expect MX and LNZ to not be effective at controlling *M.tb* growth in granulomas at concentrations close to MIC. We tested the ability of these drugs at published 1x MIC (LNZ, MX, and INH = 0.125 μg/ml; CS = 7.5 μg/ml [47]) to limit drug-susceptible (H_37_R_V_) and drug-resistant *M.tb* (using a previously published INH and EMB R clinical isolate [29]). As expected, we found LNZ and MX did not limit growth of *M.tb*, whereas INH significantly reduced H_37_R_v_, but not EMB INH resistant *M.tb*. CS showed intermediate activity (Fig S4E). Thus, results from this granuloma model were more consistent with clinical trial results in humans than published murine studies, likely due to the different granuloma architecture and cell biology observed in mice vs man [47, 48].

**Fig. 4.**
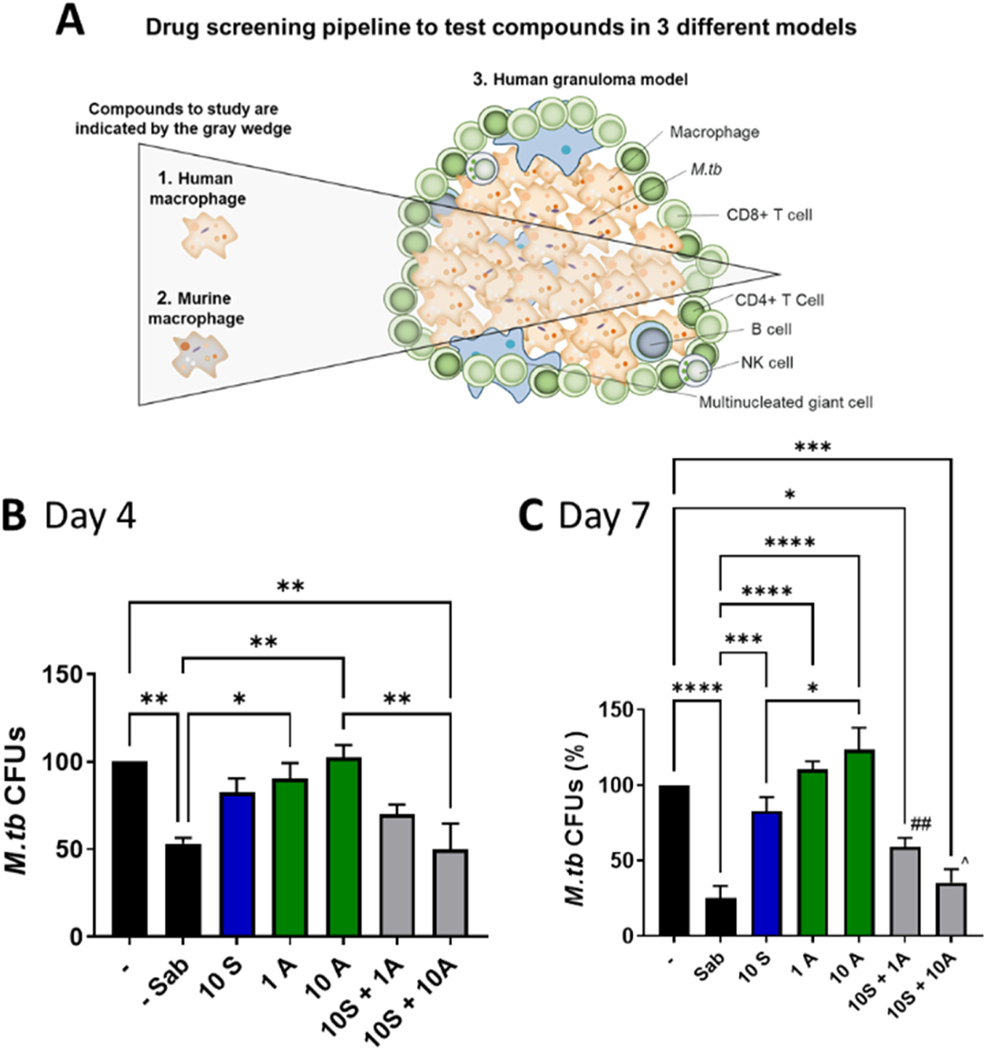
MCL-1 and BCL-2 combination therapy limits *M.tb* growth in a human granuloma model. A) Drug screening pipeline for our studies, which start with testing in primary human and murine macrophages and then proceed to testing in the human in vitro granuloma model that models human TB granulomas, a unique environment that is recalcitrant to treatment. This pipeline is used to identify and prioritize compounds that are active in these three distinct models, and so are promising compounds for TB treatment. B and C) PBMCs obtained from LTBI individuals were infected with *M.tb*, and after 1 d, treated with the BCL-2 family pan inhibitor Sabutoclax (Sab, 30 μM) the MCL-1 inhibitor S63845 (S) or the BCL-2 inhibitor ABT-199 (A), alone or in combination, at the indicated concentrations in μM. Four (B) or 7 (C) d after infection, cells were lysed and CFU enumerated. * indicates a significant difference between the indicated groups, * p < 0.05, * * p < 0.01, * ** p < 0.001, * ** * p < 0.0001, ## indicates that 10 S+ 1 A was significantly different from 1 A alone, p < 0.01, ^ indicates that 10 S+ 10 A was significantly different from the same concentration of either inhibitor alone, p < 0.05. Combination therapy was not significantly different from Sabutoclax treatment. Results are mean ± SEM of N = 5 and are expressed as % *M.tb* CFUs, relative to the untreated control.

Altogether, these data support the notion that this human in vitro granuloma model shares many key features of human TB granulomas and could serve as an important pre-clinical model (i.e., bridge to animal studies) for studying granulomatous diseases, including TB. Thus, our pipeline for identifying promising therapeutics for TB consists of first testing efficacy in human and murine macrophages to identify compounds active in macrophages, then testing those compounds in the more complex human granuloma model (Fig. 4A). In vitro granuloma models have also been proposed by others as a key tool for studying drug activity [52]. Using granuloma models is akin to using the well-established zebrafish larvae model (which is less immunologically developed and does not produce lymphocytes, but is genetically and pharmacologically tractable) to model the more complex human TB granulomas. The zebrafish model has been used to study HDTs, including efficacy of the general corticosteroid dexamethasone [53,54].

### 3.5. Combination of MCL-1 and BCL-2 inhibitors reduce M.tb growth in human in vitro granuloma structures

We had previously shown that MCL-1 inhibitors that significantly reduce *M.tb* growth in macrophages did not effectively reduce *M.tb* growth in our pre-clinical granuloma model, whereas inhibition of MCL-1, BCL-2, and BCL-X_L_ with pan MCL-1 family inhibitors did [15], suggesting that these general inhibitors more effectively penetrated the granuloma structure and/or that inhibition of MCL-1, BCL-2, and BCL-X_L_ is required when the bacteria are in multicellular complexes. Due to the key role of BCL-X_L_ in platelet apoptosis, and the adverse effects this has caused in clinical trials, we queried whether inhibition specifically of MCL-1 and BCL-2 would be sufficient to reduce *M.tb* growth in these structures. We confirmed that the granulomas remained intact during the duration of the experiment by microscopy, and although the structures remained intact, as expected, S63845 + ABT-199 reduced viability of cells in the granulomas, in a concentration and time dependent manner (Fig S4F). The size of the trypan blue-positive cells suggests that S63845 + ABT-199 may induce cell death in not just macrophages, but also lymphocytes. We also noticed a low level increase in trypan blue-positive cells in the untreated granulomas over time, with more trypan blue-positive cells at day 7 than day 4, which is expected based on our previous observations [20]. Based on the trypan blue results, we assessed CFUs in granulomas at both day 4 (Fig. 4B) and 7 (Fig. 4C) post infection. In all instances, there were trypan blue-negative cells in the granulomas, indicating that not all cells exhibited membrane damage. This is in contrast to treatment with Triton, which disaggregated the granulomas and resulted in > 99% trypan blue-positive cells. CFU results at both time points indicate significant control of *M.tb* growth in granulomas, at days 4 and 7. Specific inhibition of MCL-1 and BCL-2 with 10 μM S63845 + 1 μM ABT-199 reduced *M.tb* burden in granuloma structures by 29.93 ± 5.36% at day 4 and 41.12 ± 6.24% at day 7, and 10 μM of each inhibitor reduced *M.tb* burden by 49.99 ± 14.60% at day 4 and 64.61 ± 8.92% at day 7, to a similar extent as the pan inhibitor Sabutoclax (which reduced *M.tb* growth by 46.98 ± 3.40% and 64.61 ± 8.92% at days 4 and 7, respectively). Inhibition of MCL-1 or BCL-2 alone did not significantly reduce *M.tb* burden (Fig. 4B,C). Taken together with our previous results, this suggests that inhibition of both MCL-1 and BCL-2 is required to reduce *M.tb* growth in granulomas, and that targeting both MCL-1 and BCL-2 is a potential promising option for HDT for TB.

### 3.6. Combination use of newer phase I/II clinical MCL-1 + BCL-2 inhibitors reduces drug susceptible and resistant M.tb growth in human macrophages and granuloma structures

**Fig. 5.**
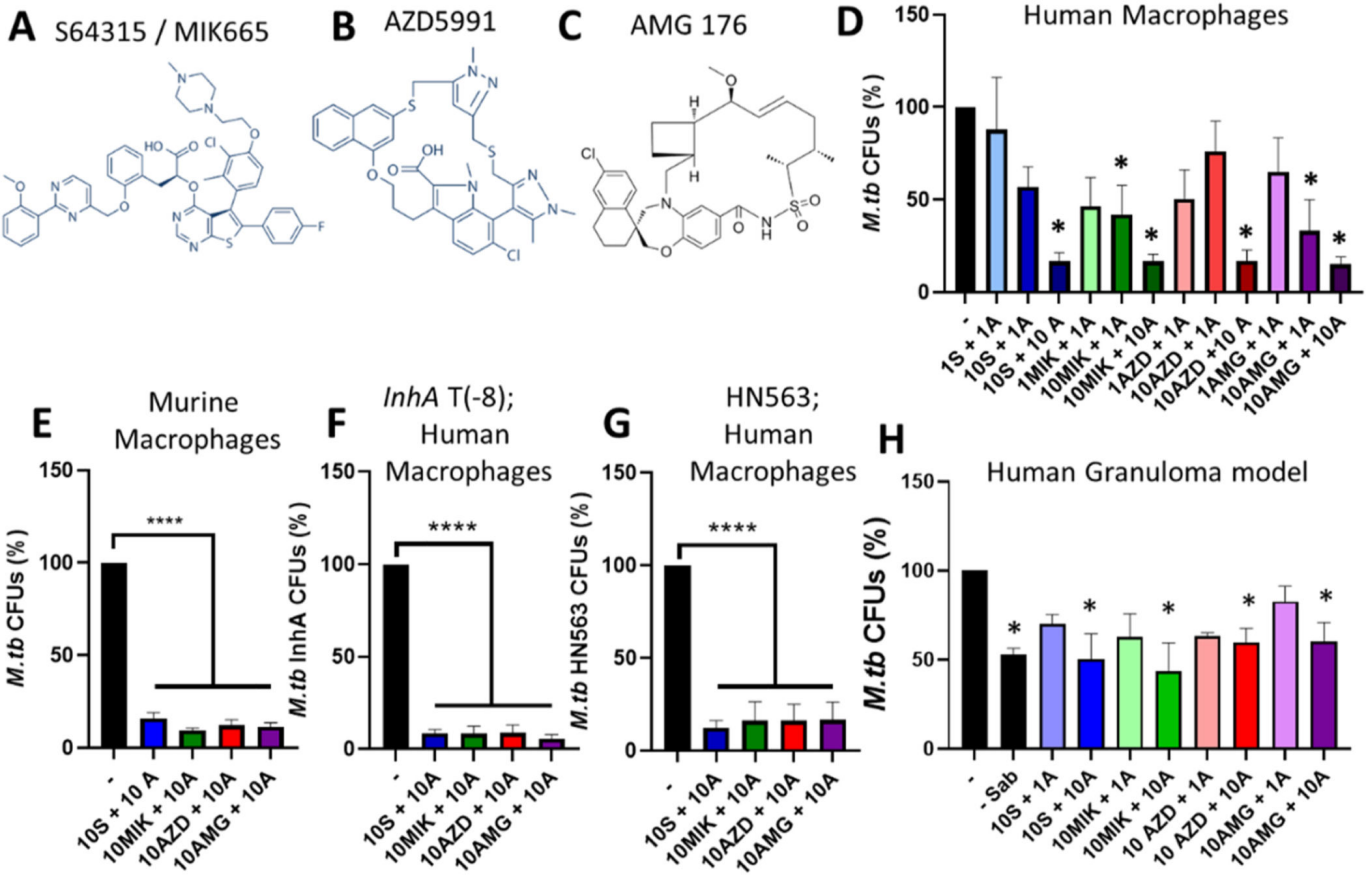
MCL-1 and BCL-2 combination therapy with inhibitors in clinical trials limits *M.tb* growth in macrophages and a human granuloma model. A-C) Chemical structures of the MCL-1 inhibitors S64315/MIK665 (A), AZD5991 (B), and AMG 176 (C), obtained from the manufacturer’s website: MIK665 and AZD5991 were reproduced from Selleckchem (https://www.selleckchem.com, accessed 2/16/23), and AMG 176 reproduced from MedChemExpress (https://www.medchemex-press.com/AMG-176.html, accessed 2/16/23) D-G) MDMs (D,F,G) or BMDMs from BALB/c mice (E) were infected with drug susceptible *M.tb* (D, E: H_37_R_v_) or drug resistant *M.tb:* InhA T(−8)(F: INH R) or HN563 (G: RIF + INH R) then treated with the BCL-2 inhibitor ABT-199 (A) and the MCL-1 inhibitors S64315/MIK665 (MIK), AZD5991 (AZD), or AMG 176 (AMG) at the indicated concentrations (μM). After 4 days, cells were lysed and CFUs enumerated. H) Human PBMCs were infected with *M.tb* at MOI 1, and after 1 d, treated with the BCL-2 pan inhibitor Sabutoclax (Sab, 30 μM), the BCL-2 inhibitor ABT-199 (A) + the MCL-1 inhibitors S64315/MIK665 (MIK), AZD5991 (AZD), or AMG 176 (AMG) at the indicated concentrations (μM). After 3 days, cells were lysed and CFU enumerated. D-H) Results are % *M.tb* CFUs, relative to the untreated control, and are the mean ± SEM of at least three independent experiments. * indicates a significant difference from untreated control, * p < 0.05, * ** * p < 0.0001.

Since we started this study, newer more potent MCL-1 specific inhibitors have been developed and advanced to clinical studies as cancer therapeutics, including S64315/MIK665 (a derivate of S63845), AMG 176, and AZD5991 (Fig. 5A-C) [16–18]. All three inhibitors were developed through structure-guided studies and specifically bind MCL-1 with K_i_ values in the pM range. They (or a similar analog in the case of AMG 176) were confirmed to inhibit MCL-1 binding to BAK/BAX and their induction of apoptosis requires BAX/BAK, confirming on-target actions. They are efficacious at reducing tumor burden in murine models and are being tested in phase I and II clinical trials, alone and in combination with BCL-2 inhibitors [11,16–18]. We were interested in determining if these MCL-1 inhibitors, similar to S63845, would act in concert with ABT-199 to reduce *M.tb* growth. To assess this, *M.tb-*infected macrophages were treated with ABT-199 + MIK665, AZD5991, AMG 176, or S63845 for comparison purposes. All four MCL-1 inhibitors in combination with ABT-199 significantly reduced *M.tb* burden in human macrophages, in a concentration dependent manner, with 10 μM ABT-199 + 10 μM MIK665, AZD5991, AMG 176, or S63845 significantly reducing *M.tb* growth by over 80% (Fig. 5D). When we combined a lower concentration of ABT-199 (1 μM) with 10 μM MIK665 or AMG 176, we observed significantly reduced *M.tb* growth by 57.77 ± 15.52% (middle green bar) and 66.77 ± 16.99% (middle purple bar), respectively; suggesting that MIK665 and AMG 176 may be more potent than S63845 or AZD5991 although there were no statistically significant differences between the different inhibitors.

Since AZD5991 and AMG 176 bind murine MCL-1 less efficiently than human MCL-1, we determined if these inhibitors would reduce *M.tb* growth in murine macrophages. As seen with S63845, ABT-199 + MIK665, AZD5991, or AMG 176 significantly reduced *M.tb* burden in murine macrophages, by over 80% (Fig. 5E). This was expected, since these inhibitors reduce tumor burden in mice [16–19]. We next confirmed that similar to S63845 ABT-199, these newer MCL-1 inhibitors + ABT-199 would reduce growth of drug resistant *M.tb* in human macrophages. As expected, treating macrophages with ABT-199 + MIK665, AZD5991, or AMG 176 was as effective against drug resistant *M.tb* as drug susceptible *M.tb,* and significantly reduced drug resistant *M.tb* growth by at least 80% (Fig. 5D versus F,G). We next determined if combination therapy with ABT-199 + MIK665, AZD5991, or AMG 176 would limit *M.tb* growth in the more complex human granuloma structures. Similar to S63845, MIK665, AZD5991, or AMG 176 + 1 μM ABT-199 reduced *M.tb* growth by 20–40% and S63845, MIK665, AZD5991, or AMG 176 + 10 μM ABT-199 significantly reduced *M.tb* growth by over 40%, to a similar extent as the pan inhibitor Sabutoclax after just 3 days (Fig. 5H). After 6 days, MIK665 + 10 μM ABT-199 significantly reduced *M.tb* growth by over 60% and AZD5991 or AMG 176 + 10 μM ABT-199 also reduced *M.tb* growth, although this was not statistically significant (Fig S4G), suggesting these inhibitors may be moderately less potent in a pre-clinical human granuloma model. Altogether, our studies using human and murine macrophages and a pre-clinical human granuloma model, indicate that targeting intrinsic/mitochondrial-mediated apoptosis (with inhibitors that are FDA-approved and in clinical trials for cancer) is a promising HDT approach for TB.

## 4. Discussion

Here we extend our previous work showing that specifically inhibiting MCL-1 and other anti-apoptotic BCL-2 proteins induces macrophage apoptosis and limits *M.tb* burden in human macrophages and in vitro granuloma structures [15]. For the first time, we show that specifically inhibiting MCL-1 and BCL-2 (without inhibition of other anti-apoptotic BCL-2 proteins like BCL-X_L_) to induce apoptosis of *M.tb* infected macrophages: 1) limits *M.tb* growth in both human and murine macrophages, 2) as expected from a HDT, reduces growth of drug resistant *M.tb* to a similar extent as drug susceptible *M.tb*, 3) combines with antibiotic treatment to more effectively reduce *M.tb* growth than antibiotics or MCL-1 and BCL-2 inhibitors alone and 4) reduces *M.tb* burden in a pre-clinical human granuloma model. Altogether, these results indicate that specific MCL-1 and BCL-2 inhibitors are promising HDTs for TB, which are needed to help limit this deadly disease.

In 2021, TB was the second leading cause of death, second only to COVID-19, and caused more than double the number of deaths as those caused by HIV/AIDS [1]. In 2019, there were over 1 million estimated cases of INH resistant TB and 465,000 cases of RIF resistant TB, highlighting the large number of drug resistant TB cases worldwide. Indeed, around 25% of deaths due to antimicrobial resistance are from RIF resistant TB [46]. The COVID pandemic reversed years of progress regarding TB treatment so that although incidence of drug resistant TB increased the number of people treated decreased by 15% [46]. In addition, there is a paucity of newly developed antibiotics for TB [45] and some have limited availability and high costs [46,55]. Drug resistant TB is projected to cost the global economy $16.7 trillion between 2015 and 2050 [56]. CDC estimates treatment costs for drug susceptible TB to be roughly $20,000; and this increases 9x for MDR TB ($182,000) and 28x for extensively drug resistant (XDR) TB ($568,000). Although these statistics are based on a 2014 research article, they highlight the severe costs associated with treating TB, a disease that requires months of treatment with multiple antibiotics [57,58]. A more recent study across multiple countries also suggested a roughly 10x increase in treatment costs associated with treating drug resistant vs susceptible TB [59]. Serious side effects are also increased with drug resistant TB, with 13% of patients presenting with hearing loss, 13% with hepatitis, and 11% with kidney impairment. Discouragingly, even after successful treatment for MDR TB, patients can have poor quality of life due to lung damage [45]. Thus, there is a clear need for better therapies for drug susceptible and resistant TB, and HDTs that work against drug resistant and susceptible bacteria and limit lung damage observed in TB disease, are particularly promising.

To help with development and identification of HDTs for TB, we need better predictive models that bridge cell monolayer cultures and animal studies. Such a bridge offers major advantages in terms of throughput, time and cost. In this study, we provide further evidence for the efficacy of a simple throughput human granuloma model that shares several features of human in vivo granulomas and contains the predominant immune cell types. We found that this model better recapitulates in vivo efficacy of several anti-TB antibiotics than standard mouse models. Zebrafish larvae are another example of a “bridge-type model”. Infection of zebrafish larvae, which are optically clear, with their natural pathogen *Mycobacterium marinum* provides a genetic and pharmacological tractable model that is amenable to imaging. This model has been used to study human TB immune responses, including characterizing mechanisms behind the human LTA4H genotypes that lead to altered TNFα production and susceptibility to disseminated TB meningitis [53,54]. The well-established zebrafish model has also been advanced for targeted HDT, including studying the efficacy of the general corticosteroid dexamethasone on TB meningitis [53,54]. Akin to using smaller and less immunologically developed zebrafish larvae to model the more complex human TB, we used an vitro *M.tb*-infected granuloma model generated with primary human immune cells. Benefits with this pre-clinical granuloma model include: it is generated with virulent and clinical *M. tb* strains and human cells (obviating any concerns over species specificity), this model contains the primary cell types in human TB granulomas including macrophages and lymphocytes (which are lacking in zebrafish larvae), it effectively predicts human clinical antibiotic efficacy studies (more so than standard C57BL/6 or BALB/c mice, where granuloma architecture is different), and in regards to animal studies it is less costly, requires less time, and does not have the ethical concerns associated with animal studies [22]. Similar in vitro granuloma models have been used as a drug screening platform by others [52]. Thus, we believe that our results that specific inhibition of MCL-1 plus BCL-2 reduces *M.tb* growth in a human granuloma model indicate that this is a potentially promising approach for TB treatment.

Our approach targeting intrinsic/mitochondrial based apoptosis in human macrophages and a pre-clinical human granuloma model, together with Pellegrini’s work targeting extrinsic apoptosis in vitro and in vivo in mice [10], indicate that inducing apoptosis is a promising and viable HDT approach to control TB, that works against both drug susceptible and drug resistant *M.tb*. There are mechanistic reasons for how apoptosis can control *M.tb* growth while cells undergo the process of programmed cell death. For example, activation of caspases involved in apoptosis leads to processing of factors that induce phagocytosis, which should help clear pathogens [39]. Indeed, efferocytosis of *M.tb-*infected macrophages reduces *M.tb* growth [41]. In addition, apoptosis is suggested to increase immune cell trafficking to lymph nodes, antigen presentation, and T cell activation, which would also contribute to improved control of *M.tb* [60]. This is in contrast to induction of other types of cell death like necrosis or ferroptosis, which are associated with increased *M.tb* growth in vitro and in mice [8,40]. Although a novel approach for TB treatment, inhibiting anti-apoptotic BCL-2 family members has been heavily studied as an approach to cancer therapy. To this end, BH3 mimetics have been developed. BH3 mimetics mimic the BH3 domain of the pro-apoptotic BH3 proteins (like BIM, PUMA, NOXA) and bind the anti-apoptotic BCL-2 proteins to inhibit the anti-apoptotic proteins and instigate apoptosis. The first BH3 mimetic to advance to clinical trials was the pan inhibitor ABT-263/navitoclax. However, these trials were halted due to on-target reduction in platelet counts, since this pan inhibitor inhibited BCL-2, BCL-W, and BCL-X_L_, the latter of which critically regulates platelet apoptosis [11]. The highly specific BCL-2 inhibitor ABT-199/venetoclax [with three orders of magnitude difference in K_i_ between BCL-2 (K_i_ < 0.010 nM) and BCL-X_L_ (K_i_ = 48 nM) or BCL-W (K_i_ = 245 nM)] was described in 2013 and in vitro and in vivo studies with rodents suggested this would be efficacious in the clinic without platelet loss and a therapeutic window for targeting cancer cells without adverse events could be achieved [38]. Indeed, ABT-199, which does not target BCL-X_L_, does not lead to platelet loss, and is now FDA approved for treatment of various hematological malignancies and was awarded ‘breakthrough designation’ by the FDA in 2017 [11,61].

Similar to BCL-2, MCL-1 is highly expressed in a range of cancer cells. Development of BH3 mimetics that bind MCL-1 have lagged behind development of BCL-2 specific inhibitors due to the much smaller and less flexible BH3 binding pocket in MCL-1 [62]. Among the early MCL-1 specific inhibitors described are A-1210477 and MIM-1 [34,63], which we showed induced apoptosis to control *M.tb* infection in human macrophages [15]. However, there are no reported animal studies for these compounds. The first potent and MCL-1 specific inhibitor described in animal studies was S63845 and this was efficacious in rodent models at reducing tumor burden, without adverse events [19]. A newer derivative of S63845, S64315/MIK665 was described in 2020 with improved potency and efficacy in mice [16]. Additional MCL-1 specific inhibitors, including AMG 176 and AZD5991, were described in 2018 and shown to be efficacious at reducing tumor burden in murine models [17,18] and are in clinical trials for lymphoma, multiple myeloma (MM), and acute myeloid leukemia (AML) [62]. Excitingly, we found that inhibition of BCL-2 with the FDA approved ABT-199 along with MCL-1 inhibition with inhibitors in clinical trials and in the preclinical stage, significantly limits *M.tb* growth in human and murine macrophages, and a pre-clinical human granuloma model.

Some cancers have been refractory towards ABT-199 treatment, and a proposed reason for this is increased MCL-1 expression [11,62]. Thus, similar to our approach for limiting *M.tb* in a granuloma model, other labs have queried if specifically inhibiting MCL-1 and BCL-2 would limit tumor burden of cells that highly express both BCL-2 and MCL-1 [11, 17–19,36,64–66]. Indeed, multiple studies assessing administration of these inhibitors, including on the same day, reported that specifically inhibiting MCL-1 and BCL-2 was effective at reducing tumor burden, without overt toxicity concerns [17–19,36,64–66]. A few publications specified that inhibitors were added on different days [36], or sequentially [18,66]. MCL-1 specific inhibitors MIK665, AMG 176, AZD5991 have advanced to phase I/II clinical trials for cancer therapies, some in conjunction with the BCL-2 inhibitor ABT-199 (rev in: [62]). One clinical trial is administering S64315/MIK665 2–4 h after ABT-199, the other trials did not specify if the inhibitors would be added sequentially or not. Thus, based on this literature, we anticipate that administering the compounds within a couple hours of each other would not cause significant toxicity in the case of *M.tb* infection. Since pharmacodynamics and tolerability are already known and/or being studied for the combinations of inhibitors that we studied, we similarly expect that our findings in macrophages and the pre-clinical granuloma model, will translate well to animal models (including non-human primates) and (more importantly) to human TB granulomas.

## 5. Conclusions

Altogether, we show that MCL-1 and BCL-2 are promising targets for HDT to control drug susceptible and resistant TB. Since most of the tested inhibitors are already in phase I/II clinical trials for cancer therapies, we are hopeful that they can be repurposed for TB therapy quicker than a newly developed drug. We posit that MCL-1 and BCL-2 inhibitors could be administered on the same day, or alternative days to reduce toxicity, in conjunction with antibiotics at the beginning of therapy (induction phase) to more quickly and effectively reduce *M.tb* burden. This approach may enable shortening of treatment duration to increase compliance, reduce development of drug resistance, and since apoptosis is associated with improved antigen presentation and reduced inflammation, could also boost the immune response while limiting damaging inflammation associated with TB disease; thereby providing a multipronged benefit to TB treatment, for both drug susceptible and resistant infections.

## Supplementary Material

1

2

3

## Data Availability

All data are included in the manuscript.

## Abbreviations:

AML: acute myeloid leukemia
ATCC: American Tissue Culture Collection
BMDM: bone marrow-derived macrophage
CFU: colony forming unit
CS: cycloserine
DMSO: dimethyl sulfoxide
HDT: host-directed therapy
HI-FBS: heat inactivated-FBS
IACUC: Institutional laboratory Animal Care and Use Committee
IGRA: IFNγ release assay
IRB: Institutional Review Board
INH: isoniazid
LTBI: latent TB infected
LNZ: linezolid
MDM: monocyte-derived macrophage
MDR: multidrug-resistant
MIC: minimal inhibitory concentration
MM: multiple myeloma
MX: moxifloxacin
PARP: poly ADP-ribose polymerase
PBMC: peripheral blood mononuclear cell
Pen/Strep: penicillin/streptomycin
PFA: paraformaldehyde
RIF: rifampicin
RLU: relative luminescence unit
TB: Tuberculosis
TNFR: tumor necrosis factor receptor
TST: tuberculin skin test
XDR: extensively drug resistant
