# Supplementary material for: Combination of MCL-1 and BCL-2 inhibitors is a promising approach for a host-directed therapy for tuberculosis
Source: Biomed Pharmacother. Author manuscript; Available in PMC 2024 Feb 5. (PMC10841846; doi:10.1016/j.biopha.2023.115738)
Supplement: 1 [file NIHMS1944570-supplement-1.docx]

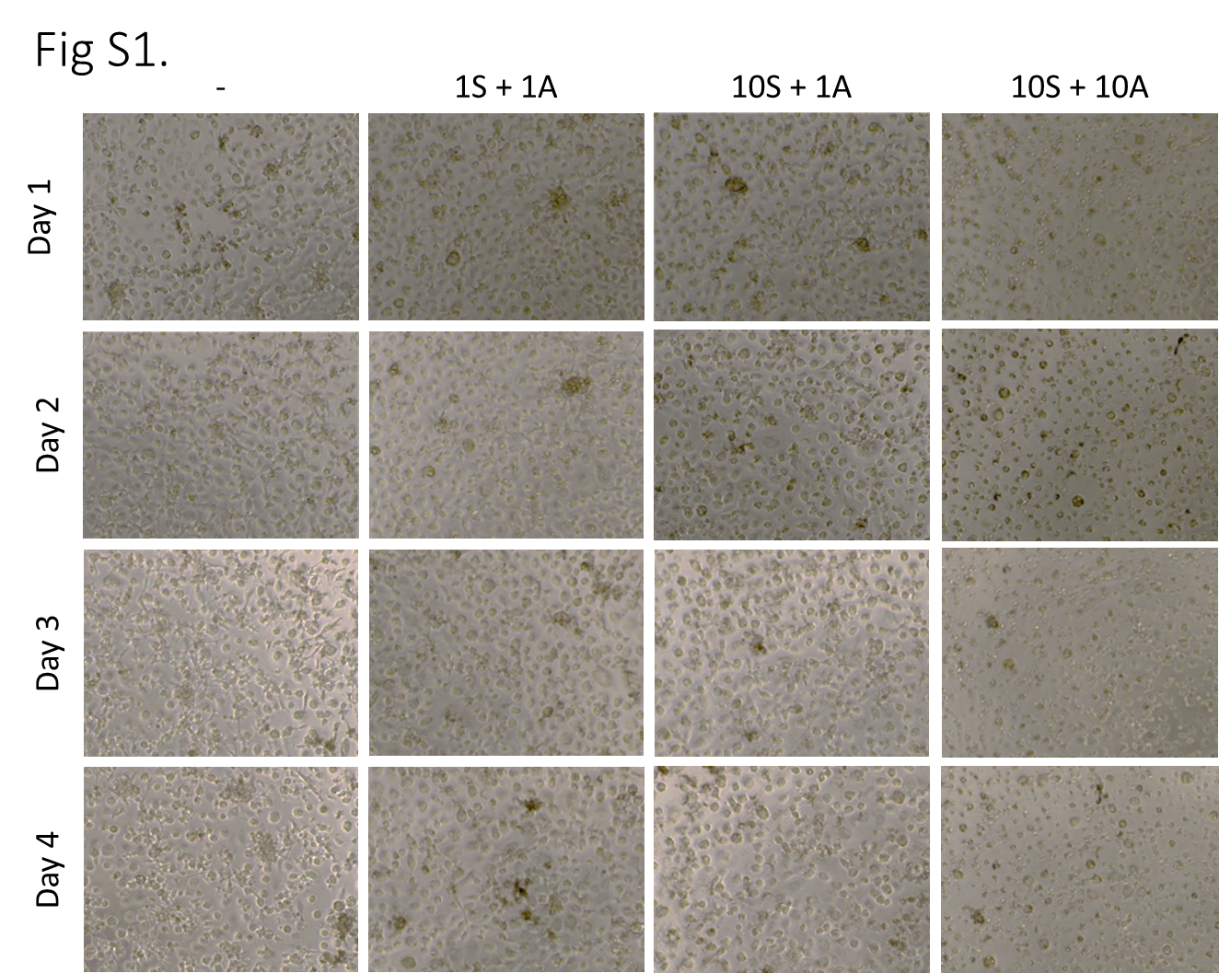


**Fig S1. MCL-1 and BCL-2 combination therapy does not disrupt macrophage monolayers, but does induce subtle morphological changes characteristic of apoptosis in *M.tb-*infected human macrophages.** MDMs were infected with *M.tb*, then treated with MCL-1 inhibitor S63845 (S) + BCL-2 inhibitor ABT-199 (A) at the indicated concentrations (μM). Cell micrographs were acquired daily. Representative images of at least four independent experiments.

**
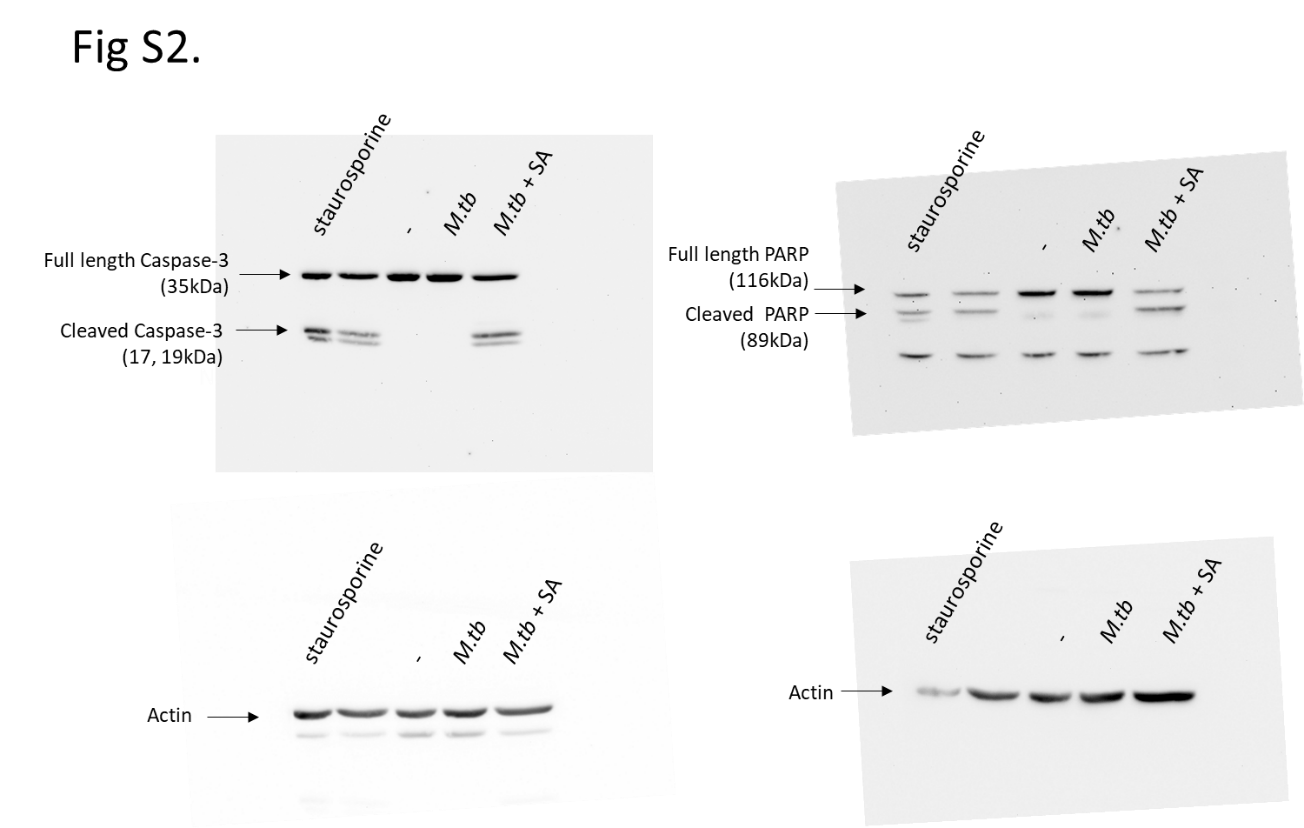
**

**Fig S2. MCL-1 and BCL-2 combination therapy induces apoptosis in *M.tb* infected human macrophages.** MDMs were infected with *M.tb*, then treated with 10μM MCL-1 inhibitor S63845 + 10μM BCL-2 inhibitor ABT-199. After 4 h, protein lysates were collected for Western blotting. Representative blot of three independent experiments, shown are uncropped blot of those shown in Fig 2D. 5 µM staurosporine serves as a positive control for apoptosis.


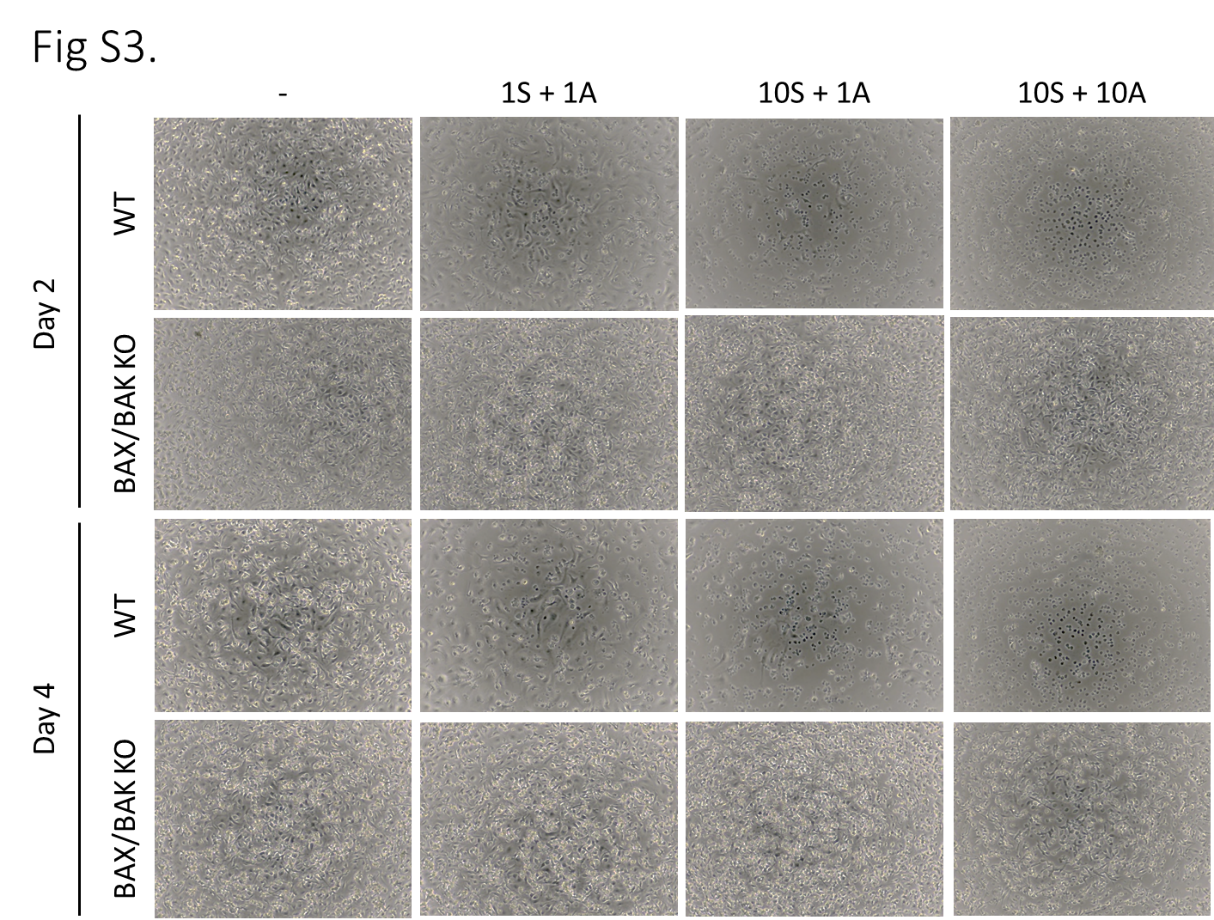


**Fig S3. MCL-1 and BCL-2 combination therapy does not disrupt the macrophage monolayer but does induce subtle morphological changes characteristic of apoptosis in a BAX/BAK-dependent manner in *M.tb-*infected murine macrophages.** BMDMs from BAX+BAK KO or control (WT) mice were infected with *M.tb*, then treated with S63845 + ABT-199 (S + A) at the indicated concentrations (μM). Cell micrographs were acquired daily. Representative images of three experiments after 2 and 4 days of infection.


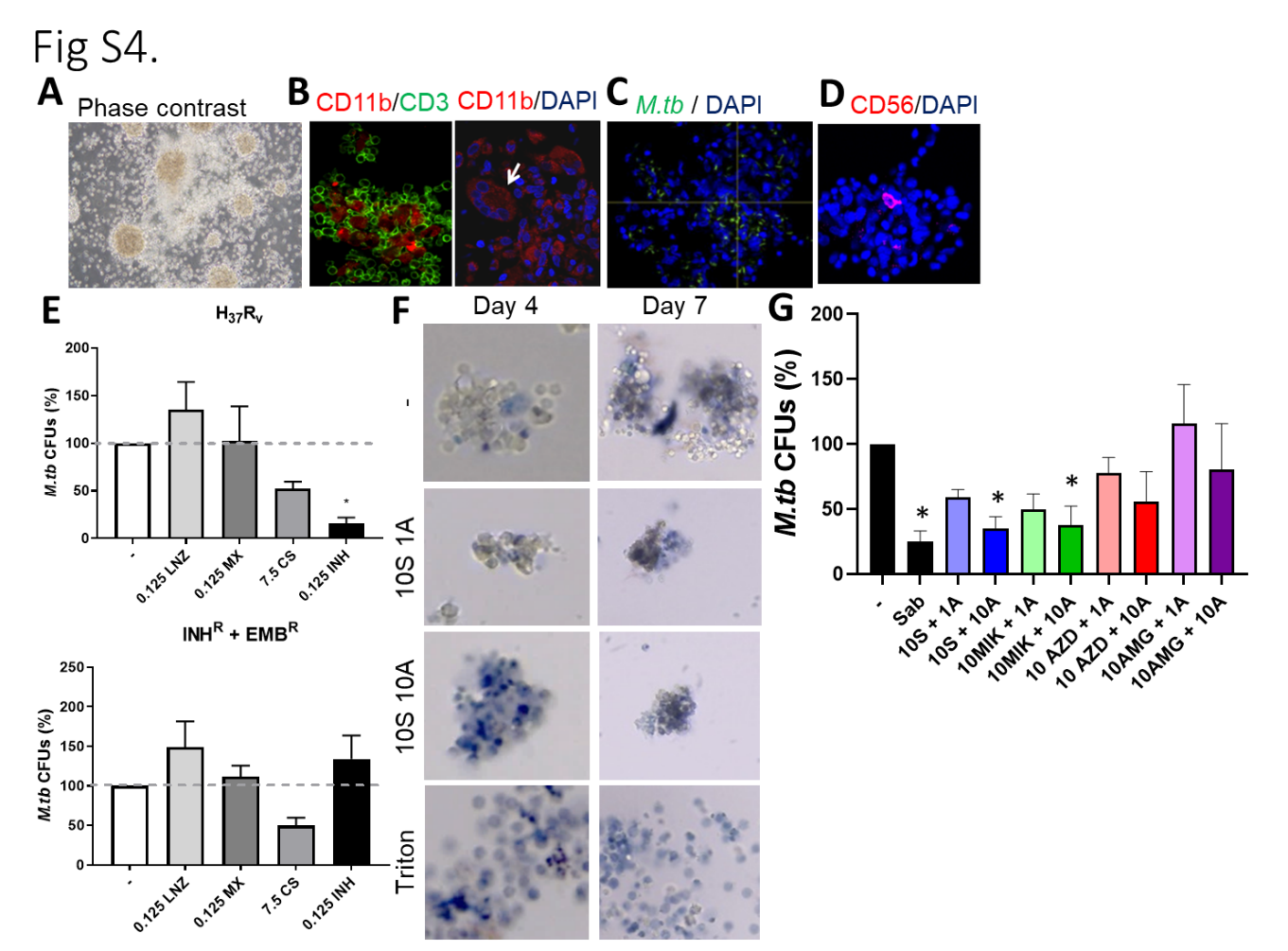


**Fig S4. An in vitro human granuloma model.** PBMCs obtained from LTBI individuals were infected with *M.tb*, resulting in the formation of granuloma structures by day 4 post infection. **A)** Phase contrast image. **B & C)** Granuloma structures contain macrophages (CD11b+, red), T cells (CD3+, green), and multinucleated giant cells (CD11b+, red, white arrow) **(B)** and intracellular bacteria (green) **(C).** Nuclei were stained with DAPI (blue). **A-C)** Images are adapted from [1]. **D)** PBMCs obtained from LTBI individuals were infected with *M.tb,* then stained with CD56 to label Natural Killer cells (pink), nuclei were stained with DAPI (blue). **E)** PBMCs were infected with *M.tb* (H_37_R_v_ or a clinical INH + EMB R strain). After 4 days, antibiotics were added at the indicated concentration (μg/ml) and CFUs were assessed after 3 more days. Results are mean ± SEM of N=3 and are expressed as % *M.tb* CFUs, relative to the untreated control. * indicates a significant difference from the untreated control, * p < 0.05. **F and G)** Human PBMCs were infected with *M.tb* at MOI 1, and after 1 d, treated with the BCL-2 pan inhibitor Sabutoclax (Sab, 30 μM), the BCL-2 inhibitor ABT-199 (A) + the MCL-1 inhibitors S63845 (S), S64315/MIK665 (MIK), AZD5991 (AZD), or AMG 176 (AMG) at the indicated concentrations (μM). After 4 or 7 days of infection structures were stained with trypan blue and imaged (**F**) or cells were lysed and CFU enumerated after 7 days of infection (**G**). **F)** Representative images of two (day 4) or 1 (day 7) independent experiments. **G)** Results are % *M.tb* CFUs, relative to the untreated control, and are the mean ± SEM of at least three independent experiments. * indicates a significant difference from untreated control, * p < 0.05.

**Movie S1. *M.tb* infected PBMCs forming in vitro granuloma like structures over time.** PBMCs obtained from LTBI individuals were infected with *M.tb* and bright field images were acquired every 6 h. The scale bar in the bottom right corner is 100 µm, the time of acquisition is indicated in the upper right corner. Live cell imaging enables viewing of these dynamic structures that change size and shape over time; with development of structures 50-100 µm in diameter forming by day 4.

**Movie S2.** **Uninfected PBMCs do not form in vitro granuloma like structures.** PBMCs obtained from LTBI individuals were left uninfected and bright field images were acquired every 6 h. The scale bar in the bottom right corner is 100 µm, the time of acquisition is indicated in the upper right corner. In the absence of infection, PBMCs remain as single cell monolayers.

**References**

[1] E. Guirado, U. Mbawuike, T.L. Keiser, J. Arcos, A.K. Azad, S.H. Wang, L.S. Schlesinger, Characterization of host and microbial determinants in individuals with latent tuberculosis infection using a human granuloma model, MBio 6(1) (2015) e02537-14. doi:10.1128/mBio.02537-14
